# TIPE1 Inhibits Breast Cancer Proliferation by Downregulating ERK Phosphorylation and Predicts a Favorable Prognosis

**DOI:** 10.3389/fonc.2019.00400

**Published:** 2019-05-22

**Authors:** Wei Hu, Chun-Mei Feng, Ling-Yun Liu, Na Li, Feng Tian, Jian-Xin Du, Yi Zhao, Xin-Xin Xiang, Kui Liu, Pei-Qing Zhao

**Affiliations:** ^1^Department of Breast and Thyroid Surgery, Zibo Central Hospital Affiliated to Shandong University, Zibo, China; ^2^Zibo City Key Laboratory of Individualized Diagnosis and Transformation of Breast Cancer, Zibo Central Hospital Affiliated to Shandong University, Zibo, China; ^3^Zibo Central Hospital Affiliated to Shandong University, Center of Translational Medicine, Zibo, China; ^4^School of Stomatology, Shandong University, Jinan, China

**Keywords:** TIPE1, breast cancer, proliferation, ERK, prognosis

## Abstract

TIPE1, which acts as a cell death regulator, has emerged as a tumor suppressor in the process of carcinogenesis. However, our recent research demonstrated that it serves as an oncogene in the pathogenesis of cervical cancer, indicating that the role of TIPE1 in carcinogenesis needs to be further evaluated. In this study, we show that TIPE1 is able to inhibit breast cancer cell growth both *in vivo* and *in vitro*. Functionally, TIPE1 inhibits cancer cell proliferation preferentially by downregulating ERK phosphorylation. Furthermore, the expression of TIPE1 is decreased in breast cancer tissues compared to matched adjacent tissues, and its expression is positively correlated with patients' lifespan. These data indicate that TIPE1 suppresses breast cancer proliferation by inhibiting the ERK signaling pathway. This study also suggests that TIPE1 could serve as a potential therapeutic target and a diagnostic biomarker for breast cancer.

## Introduction

Breast cancer is one of the most common malignant tumors in women worldwide (1). In developed countries, for example, it accounts for ~27% of all female cancers (2). Despite advances in treatment strategies involving surgery, radiotherapy, chemotherapy, endocrine therapy, and targeted therapy, patients with breast cancer still have a poor prognosis (3, 4). To date, it has been demonstrated that many molecules, such as BRCA1 (breast cancer susceptibility gene 1), HER2 (human epidermal growth factor receptor 2), and p53, are involved in the occurrence and development of breast cancer (5–7). However, the underlying mechanisms for breast cancer especially regarding its tumorigenesis remain unclear.

Tumor necrosis factor-a (TNF-a)-induced protein 8-like-1 (TNFAIP8L1 or TIPE1) is a newly identified member of the TIPE family. This family consists of four members: TNFAIP8, TIPE1, TIPE2, and TIPE3. Although previous reports have suggested that TNFAIP8, TIPE2, and TIPE3 are associated with cell proliferation, inflammation, and carcinogenesis (8–11), the biological functions of TIPE1 in physiological and pathological conditions are not fully clear. Zhang et al. showed that TIPE1 can induce hepatocellular carcinoma cells to undergo apoptosis by negatively regulating Rac1 activation (12). Moreover, very recent studies indicated that TIPE1 suppresses tumor growth, invasion, and migration in osteosarcoma, lung cancer, and gastric carcinoma (13–15). However, our recent research demonstrated that TIPE1 could promote cervical cancer proliferation, and thus preserves it as a poor prognostic factor for patients with cervical cancer (16), indicating that its biological functions for tumorigenesis remain ambiguous. Thus far, the clinical significance and biological function of TIPE1 in breast cancer have not been demonstrated. Thus, whether it serves as an oncogene or a tumor suppressor gene in breast cancer needs to be further evaluated.

In this study, we investigated that TIPE1 suppresses breast cancer cell proliferation *in vitro* and in a mouse xenograft model. Mechanistically, our findings revealed that TIPE1 inhibits cell proliferation by downregulating ERK phosphorylation. Furthermore, TIPE1 expression in breast cancer tissues is downregulated compared to matched adjacent tissues, and its expression is positively correlated with patient lifespan. Our results will provide supporting evidence to warrant further investigation of TIPE1 as a therapeutic target and a diagnostic biomarker in breast cancer.

## Materials and Methods

### Clinical Tissue Samples

A total of 52 breast cancer specimens and their counterpart adjacent tissues were collected from Zibo Central Hospital Affiliated to Shandong University and confirmed by pathological diagnosis. None of the patients received chemotherapy or radiotherapy before surgery. This study was carried out in accordance with the recommendations of the ethics committee of Zibo Central Hospital Affiliated to Shandong University. All subjects gave written informed consent in accordance with the Declaration of Helsinki. The adjacent non-tumor tissues were defined as the tissues at least 1 cm away from the tumor edge. The characteristics of the patients and the association of TIPE1 levels with clinicopathological features are summarized in Table 1.

**Table 1:** Patient's characteristics and association of TIPE1 levels with clinicopathological features in breast cancer population.

**Characteristics**	**No. of patients, *N* = 52 (%)**	***P*-value**
**AGE (years)**		
<60	39 (75%)	*P* = 0.974
≥60	13 (25%)	
**GENDER**		
Male	0 (0%)	/
Female	52 (100%)	
**TUMOR SIZE (cm)**		
<1	10 (19%)	*P* = 0.089
≥1	42 (81%)	
**LYMPH NODE METASTASIS**		
N–	18 (35%)	*P* = 0.147
N+	34 (65%)	
**DISTANT METASTASIS**		
M–	46 (88%)	*P* = 0.058
M+	6 (12%)	
**ER STATUS**		
ER–	9 (17%)	*P* < 0.001
ER+	43 (83%)	

*Differences of TIPE1 expression between experimental groups were assessed by one-way analysis of variance. Data represent mean ± SD*.

### Immunohistochemistry

Immunohistochemistry staining was performed according to the manufacturer's instructions (Zhongshanjinqiao, Beijing, China). In detail, paraffin sections were baked at 70°C for 60 min, deparaffinized in xylene, and rehydrated through graded alcohol, and then antigen retrieval was performed by microwave heating at 90°C for 3 min in citrate buffer solution (pH 6). Sections were then washed twice in PBS for 5 min and incubated with 3% hydrogen peroxide for 30 min. After washing twice with PBS, the sections were blocked with 10% goat serum for 1 h at room temperature. Finally, the sections were incubated with TIPE1 antibody (1:500 dilution; sc-82761, Santa Cruz, USA) or p-ERK antibody [1:500 dilution; #4370 (Thr202/Tyr204), CST, USA] overnight at 4°C.

### Cell Culture and Transfection

The human breast cancer cell lines MCF-7, MDA-MB-231, SKBR3, and MDA-MB-468 and the non-cancer cell line MCF10 were obtained from the Chinese Academy of Sciences Committee Type Culture Collection cell bank (Shanghai, China). Except for SKBR3, cells were maintained in DMEM (Gibco, USA) containing 10% fetal bovine serum (FBS; Gibco, USA) at 37°C with a 5% CO_2_ atmosphere. SKBR3 cells were cultivated in McCoy's 5a medium (Gibco, USA) containing 10% FBS. The shRNA against ERK, the TIPE1 lentiviral vector, the TIPE1 interference vector, and their control vectors were constructed by Genechem Company (Shanghai, China). The MCF-7 and MDA-MB-231 cell lines were seeded into six-well plates (6 × 10^5^ cells per well) and infected with the TIPE1 interference vector or the lentiviral vector and their negative controls at an MOI (multiplicity of infection) of 20.

### CCK-8 Method

Cell proliferation analysis was performed using a Cell Counting Kit-8 (CCK-8; Dojindo, Tokyo, Japan) according to the manufacturer's instructions. Briefly, cells were seeded into a 96-well plate at a density of 1 × 10^3^ cells/well and cultured at 24-h intervals for 5 days. Subsequently, the cells were treated with 10% CCK-8 solution and incubated at 37°C for 2 h. The absorbance at 450 nm was measured with a microplate reader (Thermo Fisher, USA).

### Flow Cytometry Analysis of the Cell Cycle

Cell cycle phase analysis was performed using a Cell Cycle and Apoptosis Analysis Kit (Engreen Biosystem Co, Ltd., Beijing, China). Cells were incubated in serum-free DMEM for 24 h for starvation prior to analysis and then fixed in ice-cold 70% ethanol at 4°C for 12 h. Subsequently, cells were washed with PBS and incubated with 0.1 mg/ml RNase A and 40 μg/ml propidium iodide at 37°C for 30 min. Samples were analyzed by a FACS scanner (Aria II, BD, USA).

### Colony Formation

Cells were seeded into a six-well plate at a density of 1 × 10^3^ cells/well and cultured for 10 days at 37°C in a 5% CO_2_ atmosphere. The plate was then fixed with 4% paraformaldehyde and stained with 0.1% crystal violet for 20 min. The plate was washed twice with PBS. The numbers of colonies with more than 50 cells were counted and photographed.

### Immunofluorescence

MDA-MB-231 cells transfected with an empty vector or a TIPE1 overexpression vector were dispensed onto gelatinized slides in 24-well plates at a density of 5,000 cells per well. Then, the cells were fixed for 20 min with 4% formaldehyde solution and permeabilized for 5 min with 0.5% Triton X-100 solution. After washing twice (2 × 10 min in PBS), slides were blocked with 1% bovine serum albumin (BSA) in phosphate-buffered saline containing 0.2% Tween-20, incubated overnight with the anti-p-ERK antibody [1:500 dilution; #4370 (Thr202/Tyr204), CST, USA] at 4°C, washed (2 × 10 min in PBS), incubated with an Alexa Fluor 594-labeled secondary antibody (Thermo Fisher) for an hour at room temperature, and then washed twice with PBS. A coverslip was then mounted onto the slides with Prolong Gold Antifade reagent with DAPI (Life Technologies) and washed twice (2 × 10 min in PBS). The fluorescence images of the cells were acquired with a fluorescence microscope equipped with appropriate filter combinations.

### Western Blot Analysis

Breast cancer tissues and cells were collected and lysed in ice-cold lysis buffer. Protein concentrations were measured by using a Bicinchoninic Acid Protein Assay Kit (Pierce, Rockford, USA). The protein was separated by sodium dodecyl sulfate–polyacrylamide gel electrophoresis, and then electrotransferred onto polyvinylidene fluoride membranes (Millipore, Billerica, USA). The following primary antibodies were used: anti-p-ERK [1:1,000 dilution; #4370 (Thr202/Tyr204), CST, USA], anti-ERK (1:1,000 dilution; #4695, CST, USA), anti-MEK (1:1,000 dilution; #2352, CST, USA), anti-p-MEK [1:1,000 dilution; #3958 (Ser217/221), CST, USA], anti-p65 (1:1,000 dilution; #9460, CST, USA), anti-p-p65 [1:1,000 dilution; #3033 (Ser536), CST, USA], anti-p-mTOR [1:1,000 dilution; #5536 (Ser2448), CST, USA], anti-mTOR (1:1,000 dilution; #2983, CST, USA), anti-pS6 [1:1,000 dilution; #4858 (Ser235/236), CST, USA], anti-S6 (1:1,000 dilution; #2317, CST, USA), ab85409, anti-TIPE1 (1:1,000 dilution; ab85409, Abcam, USA), and anti-GAPDH (1:2,000 dilution; Santa Cruz, USA). Next, the membranes were incubated with an HRP-conjugated anti-rabbit IgG antibody (1:5,000 dilution; CST, USA) for 1 h, followed by enhanced chemiluminescence (Santa Cruz, USA) and autoradiography.

### Animal Models

Six-week-old female nude mice were purchased from Shanghai SLAC Laboratory Animal Co. Ltd. (Shanghai, China). All mice were housed under environmentally controlled conditions. This study protocol was approved by the Animal Care and Use Committee of Shandong University. Mice were randomly divided into two groups and subcutaneously injected with approximately 1 × 10^7^ MDA-MB-231 cells containing the TIPE1 lentiviral vector or the control vector. Tumor volumes were determined every 3 days when the tumor volume reached ~80 mm^3^. Protein expression was assayed by immunohistochemical staining and Western blot analysis.

### Microarray Data

MDA-MB-231 cells transfected with the TIPE1 lentiviral vector or the control vector were collected for gene expression detection using the Affymetrix GeneChip® Human Gene 2.0 ST array. The procedures, including RNA extraction from duplicate biological replicate samples, RNA quantity assessment, cDNA synthesis, labeling of cRNA, hybridization, washing, and scanning, were performed by Genechem Company (Shanghai, China). Datasets were analyzed by Gene Ontology (GO) analysis and Kyoto Encyclopedia of Genes and Genomes (KEGG) pathway mapping.

### Statistical Analysis

All experiments were performed in triplicate. Data analysis was performed with GraphPad Prism 7.0 (GraphPad Software, San Diego, CA, USA). The statistical analyses of the experimental data were performed by using a two-tailed Student's paired *t*-test and one-way ANOVA. Survival analysis of breast cancer patients was determined by using the Kaplan-Meier method and the log-rank test. The Kaplan-Meier plotter database (www.kmplot.com) was used to determine the association between TIPE1 levels and the prognosis of breast cancer patients. A *p* < 0.05 was considered statistically significant for all tests.

## Results

### TIPE1 Can Significantly Inhibit Breast Cancer Cell Proliferation and Colony Formation *in vitro*

**Figure 1:**
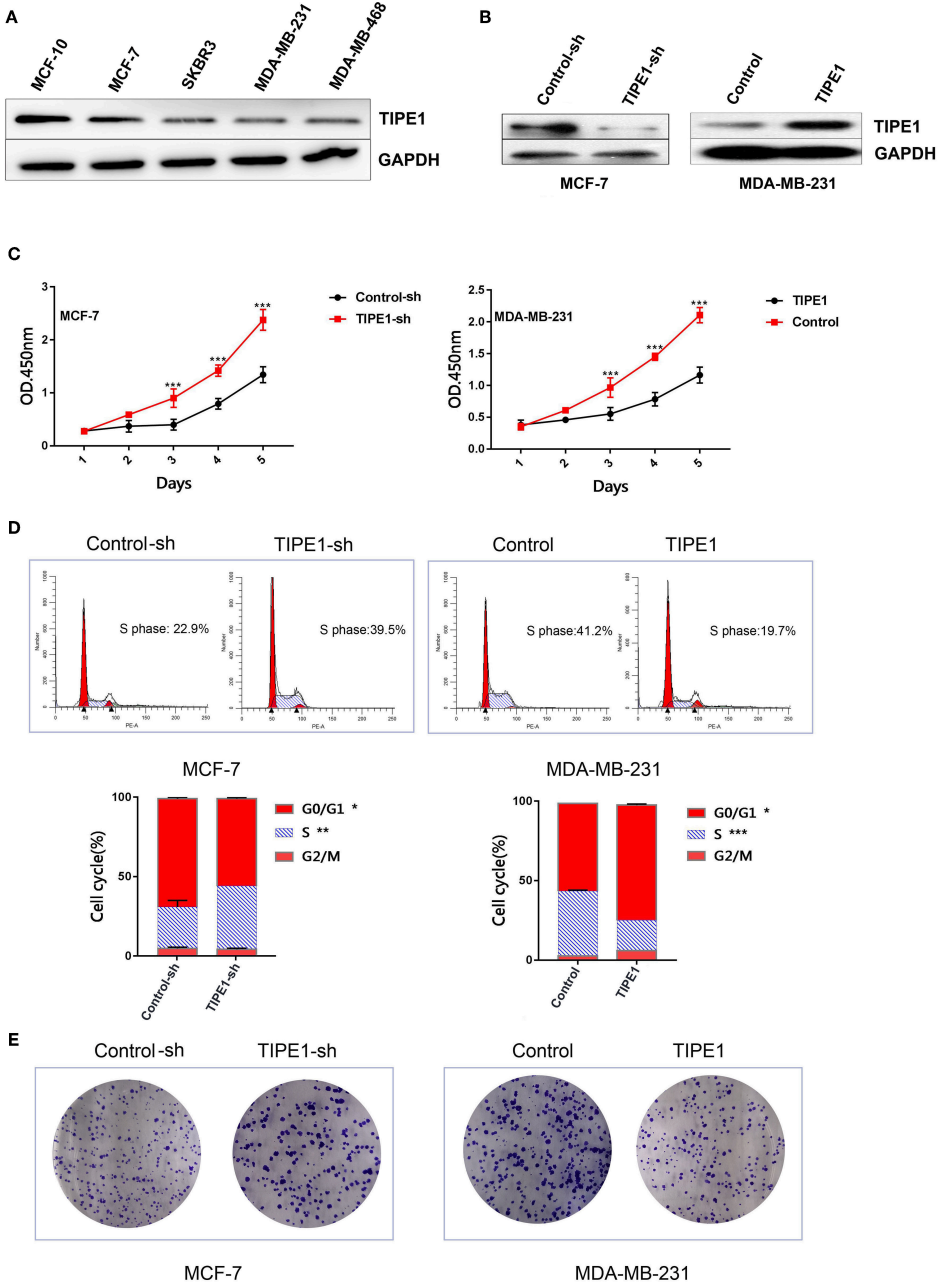
TIPE1 inhibits breast cancer progression *in vitro*. **(A)** TIPE1 expression levels in breast cancer cell lines were determined by Western blot analysis. **(B)** Cells were successfully transfected with a TIPE1 expression vector (TIPE1) or an interference vector (TIPE1-sh) in MDA-MB-231 and MCF-7 cells, as evidenced by Western blot analysis. **(C)** Analyses of cell growth curves using the CCK-8 assay. **(D)** Cell cycle was measured by PI staining. **(E)** Colony-forming assays. The values represent the mean ± SD, ^*^p < 0.05, ^**^p < 0.01, ^***^p < 0.001; the experiments were repeated three times.

To investigate the biological function of TIPE1 in breast cancer cells, we measured the expression levels of TIPE1 in breast cancer cell lines, including a non-cancer cell line (MCF10), a Her2-positive cell line (SKBR3), triple-negative cell lines (MDA-MB-231 and MDA-MB-468), and an ER-positive cell line (MCF-7). The results showed that TIPE1 expression was decreased in breast cancer cell lines compared to non-cancer cell line. Furthermore, the expression of TIPE1 in MCF-7 cells, which possess low proliferation potential, was significantly higher than that in the other cancer cells (Figure 1A). Then, we transfected the TIPE1 overexpression vector into the MDA-MB-231 cell line (low TIPE1 expression) and transfected the TIPE1 interference vector into MCF-7 cells (high TIPE1 expression) for further experiments. The knockdown or overexpression efficiency was validated using a western blot assay (Figure 1B). A CCK-8 assay was then performed to investigate the role of TIPE1 in breast cancer cell growth *in vitro*. We observed that the proliferation of cells was significantly inhibited by TIPE1 (*p* < 0.001, Figure 1C). Additionally, we investigated the effect of TIPE1 on cell cycle arrest. As shown in Figure 1D, TIPE1 significantly reduced the percentage of cells in S phase in both MCF-7 and MDA-MB-231 cells compared to the control. Consistent with the cell viability analysis, the knockdown of TIPE1 in MCF-7 cells promoted colony formation, whereas TIPE1 overexpression in MDA-MB-231 cells showed a decreased number of colonies (Figure 1E). Taken together, these results indicate that TIPE1 can significantly inhibit breast cancer cell growth *in vitro*.

### TIPE1 Suppresses the *in vivo* Growth of MDA-MB-231 Cancer Xenografts

**Figure 2:**
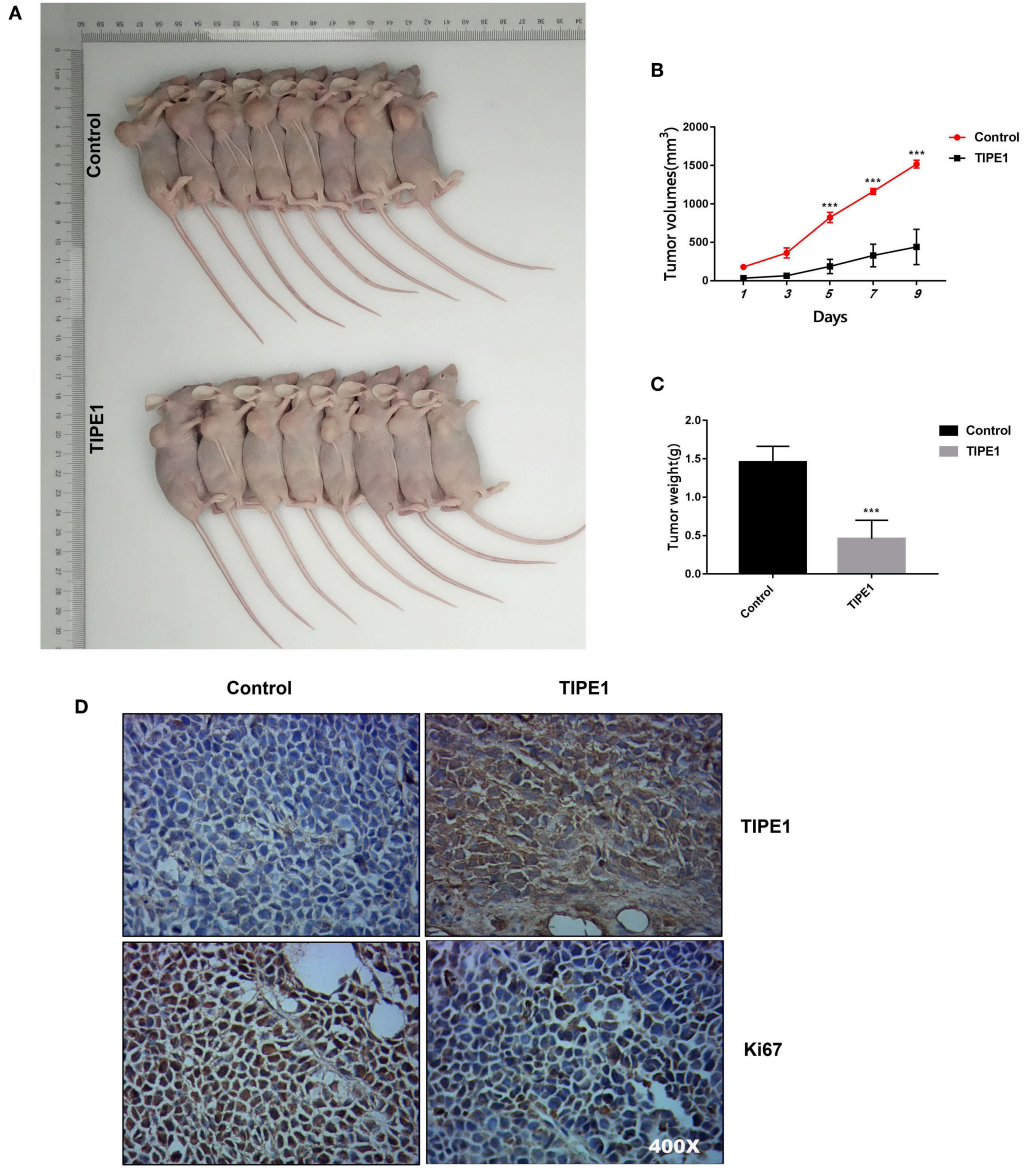
TIPE1 is a tumor suppressor for breast cancer *in vivo*. **(A)** Images of tumors in nude mice that were injected subcutaneously with MDA-MB-231 cells stably transfected with either a TIPE1 overexpression vector or a control vector. **(B,C)** Quantification of tumor volume and weight in nude mice showing a significant reduction in both tumor volume and weight after infection with TIPE1 (*n* = 8 mice) compared to control mice (*n* = 8 mice). **(D)** A negative correlation between TIPE1 and Ki67 levels in xenograft tumor tissues, as determined by immunohistochemistry. ^***^p < 0.001; the experiments were repeated three times with similar results.

The effect of TIPE1 on tumor growth was further explored in nude mice with human breast tumor xenografts. As shown in Figures 2A–C, our *in vivo* experiments showed that TIPE1 significantly reduced both tumor volume and weight compared to controls. Moreover, we also evaluated the Ki67 levels in xenografts by immunohistochemistry. As shown in Figure 2D, the Ki67 expression in the group transfected with TIPE1 was much lower compared with the group transfected with the control vector. Thus, our results show that TIPE1 exhibits significant inhibitory effect on breast tumor xenografts.

### TIPE1 Inhibits Breast Cancer Proliferation by Inhibiting ERK Phosphorylation

**Figure 3:**
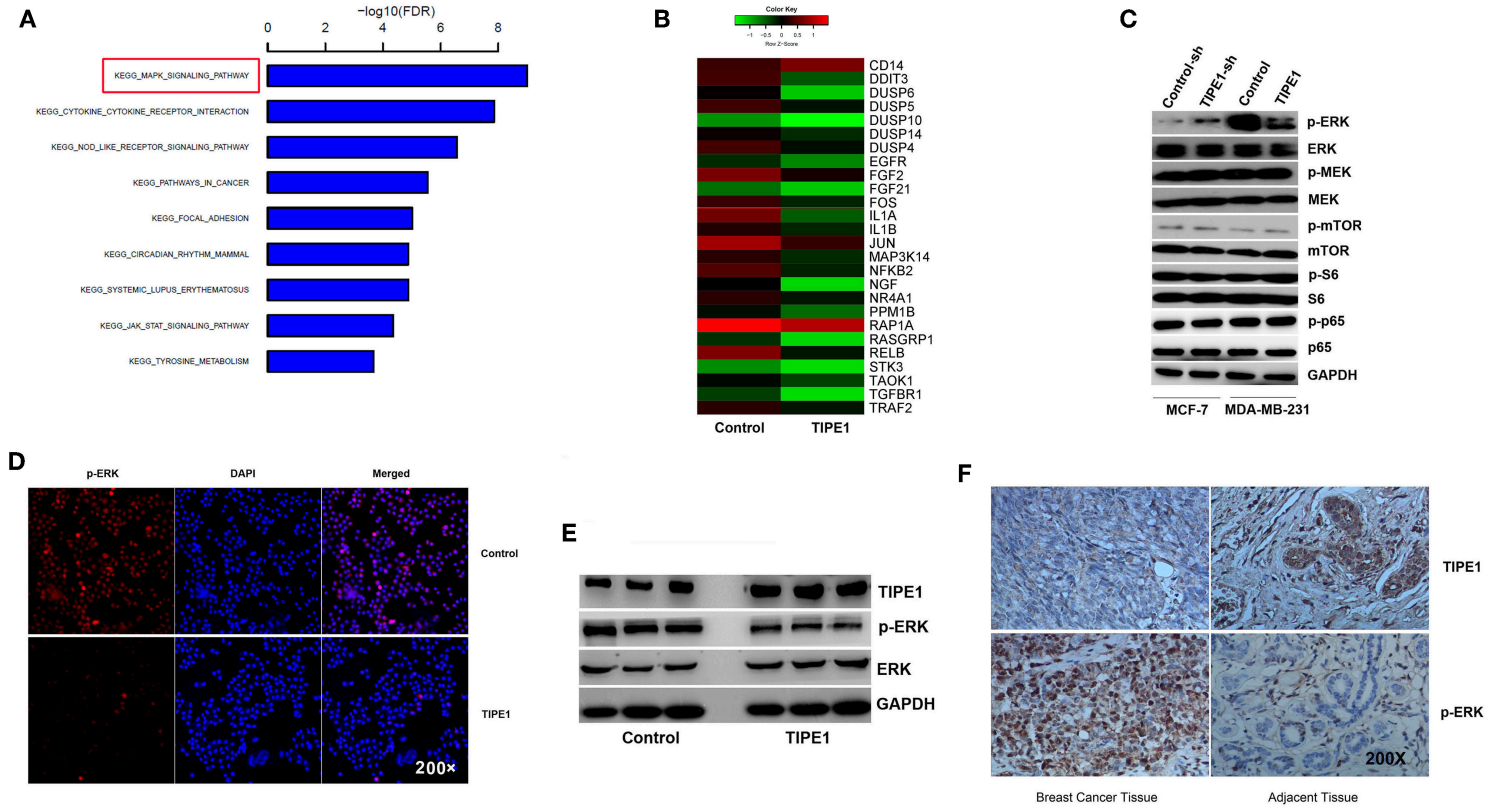
TIPE1 suppresses ERK phosphorylation. **(A,B)** Pathway analysis using Affymetrix Gene Chip hybridization demonstrates that the MAPK pathway is the top downregulated gene following forced TIPE1 expression in MDA-MB-231 cells. **(C)** TIPE1 preferentially inhibits the phosphorylation of ERK, but not that of mTOR, S6, MEK, or p65, as determined by Western blot analysis. **(D)** Immunofluorescence showing a decreased level of p-ERK after TIPE1 overexpression in MDA-MB-231 cells. p-ERK was determined by fluorescence microscopy of RFP. DAPI was used to stain nuclei. **(E)** Immunoblots showing reduced p-ERK and increased TIPE1 expression in nude mice that were injected subcutaneously with MDA-MB-231 cells stably transfected with TIPE1 compared to controls. **(F)** Immunohistochemistry analyses showing increased p-ERK and reduced TIPE1 expression in tissues from patients with breast cancer compared to adjacent non-tumor breast tissues. The experiments were repeated three times with similar results.

Furthermore, we performed gene expression and pathway analyses using Affymetrix GeneChip® Human Gene 2.0 ST arrays with MDA-MB-231 cells transfected with the TIPE1 or control vector. The results showed that the MAPK pathway was downregulated compared to the control group (Figures 3A,B). To date, several studies have shown that TIPE1 can inhibit the activation of Rac1, its downstream target p65, and the c-Jun N-terminal kinase pathway in hepatocellular carcinoma cells (12) and decrease mTOR phosphorylation by stabilizing TSC2 protein in a Parkinson's disease model (17). For further validation, we then determined the expression of these candidate genes using Western blot analysis of MDA-MB-231 cells overexpressing TIPE1 and MCF-7 cells transfected with TIPE1-sh. The results showed that, consistent with our gene chip result, TIPE1 preferentially inhibited the ERK phosphorylation (p-ERK), but not p-p65, p-mTOR, p-S6, or p-MEK, in breast cancer cells (Figure 3C). Immunofluorescence also showed a consistent phenomenon (Figure 3D). Therefore, we hypothesized that TIPE1 decreases breast cancer cell proliferation primarily through ERK signaling. To further consolidate this hypothesis, we detected the levels of ERK signaling in both clinical specimens and mouse tumor tissues. Not surprisingly, TIPE1 has a significant negative correlation with p-ERK in mouse tumor tissues (Figure 3E) and clinical specimens (Figure 3F).

### ERK Signaling Is Essential for TIPE1-Mediated Breast Cancer Cell Suppression

**Figure 4:**
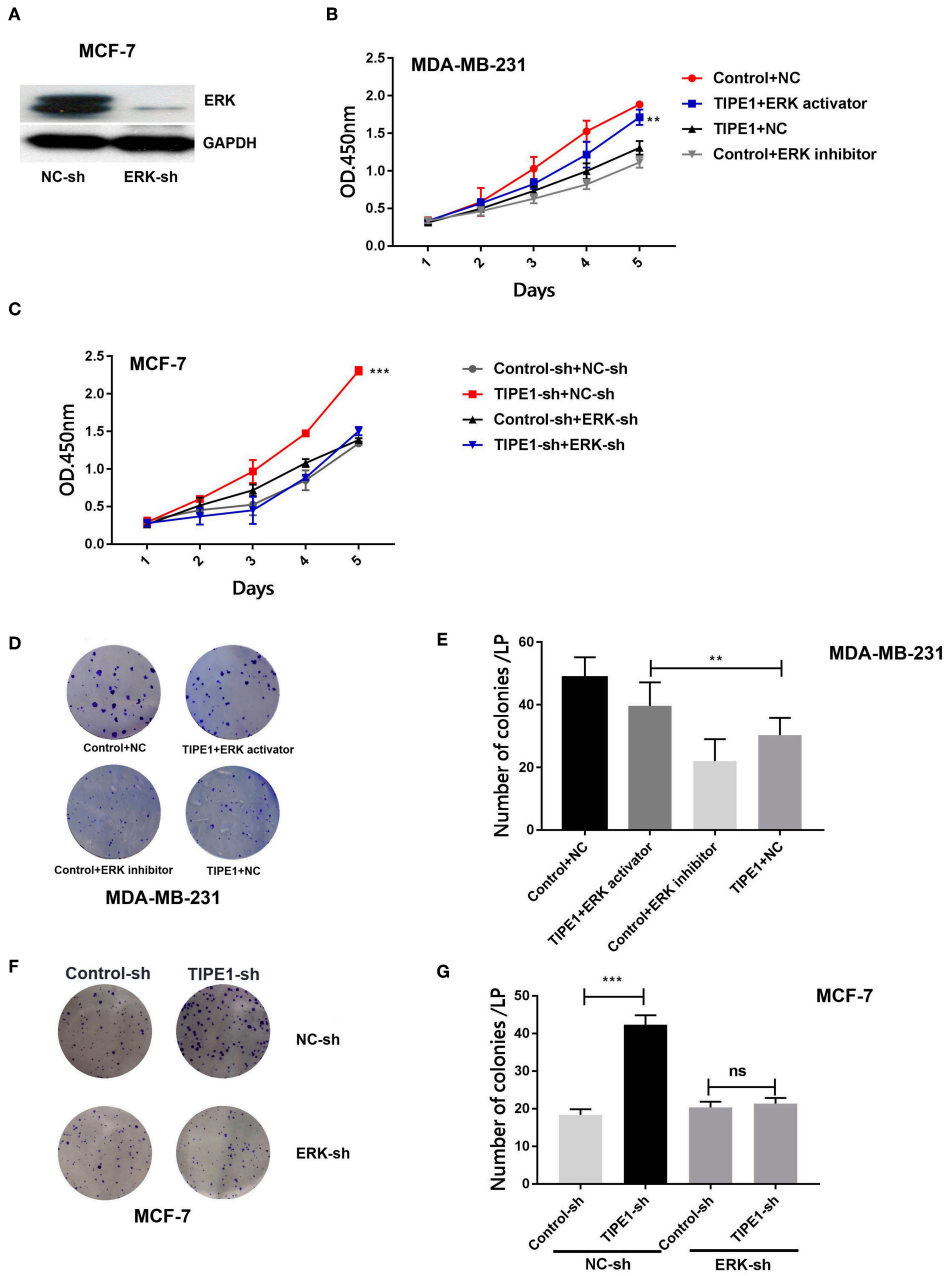
The ERK pathway is essential for TIPE1-mediated breast cancer suppression *in vitro*. **(A)** The efficiency of the ERK shRNA by Western blot analysis. MDA-MB-231 cells were cotransfected with TIPE1 and treated with the ERK pathway inhibitor or activator, and MCF-7 cells were cotransfected with the TIPE1-sh and ERK-sh, respectively, or controls. **(B,C)** CCK-8 assays at different time points were performed. **(D–G)** Colony formation assays were conducted and quantitatively analyzed. ns, no significance, ^**^p < 0.01, ^***^p < 0.001; the experiments were repeated three times.

To identify whether TIPE1 suppressed the proliferation of breast cancer cells specifically *via* the inhibition of ERK signaling, we further used the ERK shRNA and its pharmacological inhibitor and activator to perform next experiments. The efficiency of the ERK shRNA was determined by Western blot analysis (Figure 4A). MDA-MB-231 cells were transfected with the TIPE1 or control vector and then treated with the ERK pathway inhibitor (SCH772984, Selleck Chemicals, USA) or activator (sc-201242, Santa Cruz, USA). The results showed that the activation of ERK could restore cell proliferation and colony formation that are suppressed by TIPE1 in MDA-MB-231 cells (Figures 4B,D,E). Similarly, the knockdown of ERK inhibited the proliferation and clone formation rates in MCF-7 cells regardless of transfection with TIPE1-sh (Figures 4C,F,G). These data suggest that TIPE1 inhibits breast cancer proliferation preferentially by suppressing ERK activity.

### Decreased Expression of TIPE1 Is Positively Correlated With Disease Prognosis in Patients With Breast Cancer

**Figure 5:**
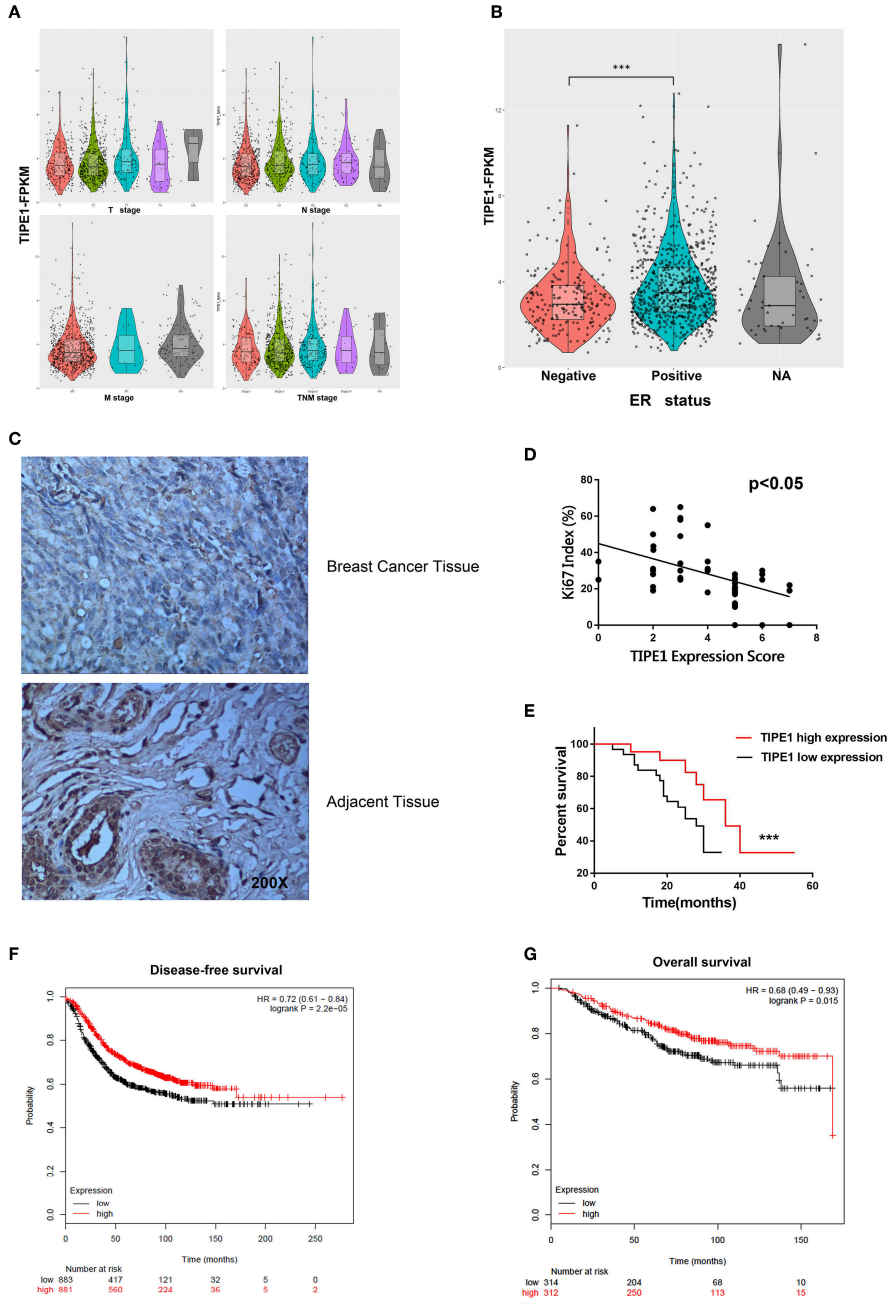
Loss of TIPE1 expression in human breast cancer positively correlates with patient survival. **(A)** TCGA dataset showing that TIPE1 expression is not associated with tumor volume or TNM stage. **(B)** TIPE1 expression is increased in the ER-positive group, as confirmed by TCGA dataset. **(C)** Immunohistochemistry assay showing reduced TIPE1 expression in tumor tissues from patients with breast cancer compared to adjacent non-tumor breast tissues (*n* = 52). **(D)** Quantification of immunohistochemistry indicates that TIPE1 and Ki67 are negatively correlated in human breast cancer tissues. **(E)** Log-rank test showing the correlation between TIPE1 expression and clinical outcome, as analyzed for overall survival. **(F,G)** The prognostic values of TIPE1 in breast cancer patients showing that the low TIPE1 expression is correlated with poor disease-free survival and overall survival in breast cancer patients [HR 95% CI = 0.72 (0.61–0.84); HR 95% CI = 0.68 (0.49–0.93), respectively], examined in www.kmplot.com. Values represent the mean ± SD; for all panels, ^***^p < 0.001.

To further confirm the roles of TIPE1 in breast cancer, we analyzed the relationship between TIPE1 expression and clinical pathology variables (Table 1). The results showed that TIPE1 expression was not associated with tumor volume or TNM stage. However, TIPE1 expression was increased in the ER-positive group, which was also confirmed by TCGA dataset (Figures 5A,B). Moreover, we compared the expression levels of TIPE1 in breast cancer specimens and their matched adjacent tissues. As shown in Figure 5C, TIPE1 was downregulated in breast cancer compared to its paired control. Further analysis indicated that the expression of TIPE1 was also negatively associated with Ki67 levels (Figure 5D), further indicating that TIPE1 suppresses cell proliferation in breast cancer. Then, we investigated whether breast cancer patients with lower TIPE1 levels had a poorer prognosis than patients with higher TIPE1 expression. Not surprisingly, the result showed that the expression levels of TIPE1 were significantly positively related to disease prognosis (Figure 5E). Consistent with these results, the prognostic values of TIPE1 in breast cancer patients were examined in an online database for prognostic analysis (Kaplan-Meier Plotter, www.kmplot.com). We found that low TIPE1 expression was correlated with poor disease-free survival and overall survival in breast cancer patients (Figures 5F,G) [*p* < 0.0001, HR 95% CI = 0.72 (0.61–0.84); *p* = 0.015, HR 95% CI = 0.68 (0.49–0.93), respectively]. These results demonstrate that decreased TIPE1 expression might serve as a prognostic factor for the outcome in patients with breast cancer.

## Discussion

Recently, members of the TIPE family have been identified as strong regulators of immune homeostasis, inflammation, and tumorigenesis (18). Although they exhibit significant domain homology, their biological functions are versatile (19). Our group documented that TIPE2 serves as a negative regulator in certain inflammatory diseases (20–22). Moreover, TIPE2 can sensitize osteosarcoma cells to cis-platin by downregulating MDR1 transcription, while TIPE1 promotes cervical cancer progression (16, 23). Thus, based on their implications in the development and progression of a variety of cancers (9, 10, 12, 24), their roles, especially regarding carcinogenesis, need to be fully evaluated.

In this study, we demonstrated that TIPE1 serves as a tumor suppressor gene in breast cancer. TIPE1 is a recently identified member of the TIPE family that acts as a cell death regulator (25). Recent studies have shown that the downregulation of TIPE1 is positively correlated with patient survival in HCC, and it serves as a novel prognostic indicator by reducing cell growth and metastasis in several cancers (13, 14, 26). However, TIPE1 mRNA is upregulated in several cancer cell lines (27), and our previous study also showed that it promotes cervical cancer proliferation (16). Thus, our research might be a noteworthy finding, since the biological functions of TIPE1 regarding tumorigenesis remain ambiguous.

Here, we showed that TIPE1 significantly decreases breast cancer cell growth both *in vivo* and *in vitro*. Unlike TIPE2, TIPE1 is expressed in various mouse tissues except for mature B and T lymphocytes and in most human carcinoma cell lines (27). For its functions regarding carcinogenesis, for example, it significantly reduces HCC cell proliferation and induces caspase-independent apoptosis by inhibiting Rac1 and its targets p65 and the c-Jun N-terminal kinase pathway (12), in contrast with our previous report in which TIPE1 promoted cervical cancer proliferation by suppressing p53 activity (16). Thus, our research can provide information to enrich its physiological and pathological functions regarding carcinogenesis.

In addition, TIPE1 was found to inhibit Rac1, and it can suppress the JNK and NF-κB pathways (19). Moreover, TIPE1 decreases mTOR phosphorylation by stabilizing TSC2 protein in a Parkinson's disease model (17). Here, we speculated how TIPE1 performs its biological functions in breast cancer. The ERK pathway plays an important role in promoting cell growth and proliferation in cancers (28). However, the underlying mechanism and correlation between TIPE1 and ERK signaling have not been elucidated. Thus, we analyzed the pathways after enforced TIPE1 expression in MDA-MB-231 cells using GeneChip arrays. The results showed that the MAPK pathway was dramatically decreased. Furthermore, we measured NF-κB, mTOR, and ERK signaling, following the overexpression or downregulation of TIPE1. The results showed that, consistent with our gene chip result, TIPE1 almost completely inhibited ERK phosphorylation (p-ERK) but not p-p65, p-mTOR, pS6, or p-MEK in breast cancer cells. This finding suggests that TIPE1 decreases breast cancer cell proliferation primarily by hampering ERK phosphorylation. To further consolidate this hypothesis, we examined the levels of ERK signaling in both clinical specimens and mouse tumor tissues. Not surprisingly, TIPE1 showed a negative correlation with p-ERK in clinical specimens and mouse tumor tissues. Furthermore, the effect of TIPE1 on the inhibition of cell proliferation and clone formation was dramatically diminished when transfected with the ERK-sh vector in MCF-7 cells. Moreover, our results further demonstrated that the activation of ERK could restore cell proliferation and colony formation that are suppressed by TIPE1 in MDA-MB-231 cells. These results indicate that TIPE1 could display ERK-dependent effects in breast cancer cell proliferation and clone formation.

We also demonstrated for the first time that TIPE1 was markedly downregulated in human breast cancer tissues. More importantly, its levels were negatively associated with Ki67, and its expression was associated with a favorable prognosis in patients with breast cancer. These results further indicate that TIPE1 functions as a tumor suppressor gene in breast cancer. Interestingly, our results showed that the expression of TIPE1 in the ER-positive cell line (MCF-7) was significantly higher than that in the other cancer cells. Consistently, our clinicopathological characteristic analyses also displayed that TIPE1 expression was increased in the ER-positive group, which was further confirmed by TCGA dataset. This phenomenon should urge us to further validate the relationship between TIPE1 and ER status in breast cancer in the future.

In summary, we demonstrated that TIPE1 suppresses breast cancer proliferation by inhibiting ERK activation. First, we investigated that TIPE1 suppresses breast cancer cell proliferation both *in vitro* and *in vivo*. Subsequent results showed that TIPE1 contributes to an inhibitory effect on ERK phosphorylation, suggesting that TIPE1 might serve as an inhibitor of breast cancer proliferation. More importantly, TIPE1 expression is downregulated in breast cancer tissues and is positively correlated with patient lifespan, indicating that it might serve as a therapeutic target and a diagnostic biomarker for breast cancer.

## Data Availability

All datasets generated for this study are included in the manuscript and/or the supplementary files.

## Ethics Statement

This study was carried out in accordance with the recommendations of the ethics committee of Zibo Central Hospital Affiliated to Shandong University. All subjects gave written informed consent in accordance with the Declaration of Helsinki.

## Author Contributions

P-QZ, KL, and WH designed the experiments, performed the experiments, analyzed the data, and wrote the paper. C-MF, L-YL, NL, FT, J-XD, YZ, X-XX, and WH performed the experiments. All authors read and approved the final manuscript.

### Conflict of Interest Statement

The authors declare that the research was conducted in the absence of any commercial or financial relationships that could be construed as a potential conflict of interest.
